# Prediction of HF-Related Mortality Risk Using Genetic Risk Score Alone and in Combination With Traditional Risk Factors

**DOI:** 10.3389/fcvm.2021.634966

**Published:** 2021-04-26

**Authors:** Dong Hu, Lei Xiao, Shiyang Li, Senlin Hu, Yang Sun, Yan Wang, Dao Wen Wang

**Affiliations:** ^1^Division of Cardiology, Department of Internal Medicine, Tongji Medical College, Tongji Hospital, Huazhong University of Science and Technology, Wuhan, China; ^2^Hubei Key Laboratory of Genetics and Molecular Mechanisms of Cardiological Disorders, Wuhan, China; ^3^Division of Cardiology, Panzhihua Central Hospital, Panzhihua, China

**Keywords:** genetic risk score, traditional risk factors, prediction, heart failure, prognosis

## Abstract

**Background:** Common variants may contribute to the variation of prognosis of heart failure (HF) among individual patients, but no systematical analysis was conducted using transcriptomic and whole exome sequencing (WES) data. We aimed to construct a genetic risk score (GRS) and estimate its potential as a predictive tool for HF-related mortality risk alone and in combination with traditional risk factors (TRFs).

**Methods and Results:** We reanalyzed the transcriptomic data of 177 failing hearts and 136 healthy donors. Differentially expressed genes (fold change >1.5 or <0.68 and adjusted *P* < 0.05) were selected for prognosis analysis using our whole exome sequencing and follow-up data with 998 HF patients. Statistically significant variants in these genes were prepared for GRS construction. Traditional risk variables were in combination with GRS for the construct of the composite risk score. Kaplan–Meier curves and receiver operating characteristic (ROC) analysis were used to assess the effect of GRS and the composite risk score on the prognosis of HF and discriminant power, respectively. We found 157 upregulated and 173 downregulated genes. In these genes, 31 variants that were associated with the prognosis of HF were finally identified to develop GRS. Compared with individuals with low risk score, patients with medium- and high-risk score showed 2.78 (95%CI = 1.82–4.24, *P* = 2 × 10^−6^) and 6.54 (95%CI = 4.42–9.71, *P* = 6 × 10^−21^) -fold mortality risk, respectively. The composite risk score combining GRS and TRF predicted mortality risk with an HR = 5.41 (95% CI = 2.72–10.64, *P* = 1 × 10^−6^) for medium vs. low risk and HR = 22.72 (95% CI = 11.9–43.48, *P* = 5 × 10^−21^) for high vs. low risk. The discriminant power of GRS is excellent with a C statistic of 0.739, which is comparable to that of TRF (C statistic = 0.791). The combination of GRS and TRF could significantly increase the predictive ability (C statistic = 0.853).

**Conclusions:** The 31-SNP GRS could well distinguish those HF patients with poor prognosis from those with better prognosis and provide clinician with reference for the intensive therapy, especially when combined with TRF.

**Clinical Trial Registration:** https://www.clinicaltrials.gov/, identifier: NCT03461107.

## Introduction

Heart failure (HF) is the final pathway of many cardiovascular problems with high morbidity and mortality (1, 2). Along with growing aging population and HF-related risk factors (e.g., hypertension, obesity, diabetes), the incidence and prevalence of HF have continuously increased (3–5). Despite effective drug treatment including β-blockers and inhibitors of the renin-angiotensin-aldosterone system, the prognosis of HF has still remained unoptimistic (4, 6).

The clinical course and prognosis of HF patients showed significantly variable among different subgroups of patients (5, 7). In view of this, a substantial amount of studies were carried out to develop the prognostic multivariable models for mortality risk stratification of HF (5, 8–12). There have been three validated and commonly used scores in chronic HF including the MECKI score, the Seattle HF Risk Model, and the MAGGIC Risk score (13–15). In these models, plenty of variables such as baseline characteristics, medical history, demographics physical exam, laboratory values, and biological markers were taken into account to develop the risk score (11, 16). Importantly, they all displayed an excellent discrimination with C statistic beyond 0.7 and could provide an accurate prediction for survival of HF (9, 13, 17). However, all these models only paid attention to conventional risk factors and ignored the importance of genetic factors in the progression of HF (1, 2). A growing body of evidence has demonstrated that hereditary factor played a vital role in the prognosis of HF (18–21). But these investigations just focused on a single variant, most of which had only modest or small effect on the mortality risk prediction of HF. Thus, it is essential to evaluate the cumulative effects of multiple loci on the mortality risk of HF and develop an HF-related genetic risk score (GRS), which could combine with traditional risk factors for the assessment of the composite risk score.

Therefore, we aim to construct a GRS for the prognosis of HF and evaluate a composite risk score comprised of both GRS and traditional risk factors in its ability to predict the mortality risk of HF.

## Methods

### Study Subjects for Whole Exome Sequencing

The study protocol conforms to the ethical guidelines of the 1,975 Declaration of Helsinki as reflected in the a priori approval by the Review Board of Tongji College of Medicine. Written informed consents were obtained from all patients before enrollment. This study is based on data from two previous studies (22, 23). Details about HF population, whole exome sequencing (WES), and bioinformatics workflow, data processing, and quality control have been described previously (22). Among our population, there are 704 patients with an LVEF value <40%, 160 patients with an LVEF value = 40–49%, and 134 patients with LVEF > 50%. The diagnosis and exclusion criteria of chronic HF have been described previously in detail (19). The composite of heart transplantation and cardiovascular death were defined as the primary end points.

### Transcriptomic Analysis and Gene Selection

Cordero et al. have conducted RNA-sequencing of 177 failing hearts and 136 healthy donor controls (23). Related data are available in GEO (accession number GSE57338). As we all know, differentially expressed genes are more likely to play a vital role in the process of HF. So we used GEO2R to compare HF and control groups to identify genes that are differentially expressed across experimental conditions. Genes with fold change (FC)>1.5 or <0.68 and adjusted *P* < 0.05 [adjusted by FDR (false discovery rate)] were selected as candidate genes for further analysis, which could also reduce the chance of overfitting the prediction model compared with involving all genes.

### Genetic Risk Score

Common single nucleotide polymorphisms (SNPs) with minor allele frequency (MAF)>0.05 in the candidate genes were extracted from our WES data. Kaplan–Meier curves were performed to evaluate the effect of above common SNPs on the prognosis of HF. Statistically significant variants were further analyzed using Cox proportional hazard to assess hazard ratios (HRs) with 95% confidence intervals (CI) for each SNP. Variants in strong linkage disequilibrium (LD) with each other (r^2^ > 0.9) were analyzed using our WES data, and only one SNP was selected as tagged SNP for the construction of GRS. Genotypes with higher mortality risk for HF were given a weighted score of 1^*^ hazard ratio (HR), while the rest were given a weighted score of 1. For each patient, the sum of the weighted scores from above SNPs were calculated and used to predict major clinical events-free survival.

### Composite Risk Score Construction

All traditional HF mortality-related variables were entered into multivariable Cox proportional hazards models together with the GRS to evaluate its independent relationship to the mortality risk of HF. The GRS was divided into thirds, and groups of low, moderate, and high risk were created with subjects in the low genetic risk of GRS as the reference. Similarly, all the continuous variables were divided into thirds and into groups of low, moderate, and high risk. The corresponding beta coefficients for each variable were then used to create a weighted composite score consisting of those variables showing a significant association with the prognosis of HF. The beta coefficients from each category were used for the continuous variables categorized. The composite risk score was divided into thirds and further into groups of low, moderate, and high risk and then analyzed using Cox proportional hazards models.

### Statistical Analysis

Statistical analyses were performed with Statistical Package for the Social Sciences (SPSS), version 13.0, and R version 3.5.0. Data were presented as mean ± standard deviation (SD) for continuous variables and median [interquartile range (IQR)] or numbers (percentages) for categorical or dichotomous variables. Linkage disequilibrium was calculated using Haploview version 4.1. Kaplan–Meier curves and the Cox proportional hazards regression model were used to assess the association of GRS and the composite risk score with the prognosis of HF. Statistical significance were compared by either unpaired or paired, two-tailed Student's *t-*test or one-way ANOVA followed by Bonferroni's *post-hoc* test, where appropriate.

Traditional risk factors for mortality risk of HF were defined as age, gender, hypertension, diabetes, smoking, LVEF, hemoglobin, NT-proBNP (logarithmic transformation of NT-proBNP is used in order to minimize the effect of extreme values), serum creatinine, potassium, sodium, systolic blood pressure, and diastolic blood pressure. Receiver operating characteristic (ROC) curve analysis with MedCalc 11.5 (http://www.medcalc.be/) was performed to compare the discriminant power of traditional risk factors, GRS, and the composite risk score. All comparisons were two-sided, and *P* < 0.05 was considered as significant.

## Results

### Subjects Characteristics

A total of 1,000 chronic HF patients (787 patients with dilated cardiomyopathy and 213 patients with ischemic cardiomyopathy) were recruited, in which we completed the follow-up with 998 patients finally. During the follow-up, 260 primary endpoint events occurred. Detailed characteristics of the participants are listed in Table 1.

**Table 1:** Baseline characteristics of population with whole exome sequencing.

**Characteristics**	**Sequencing population (*N =* 1,000)**
Men	743
Age, years	57.00 ± 14.19
LVEF (%)	34.55 ± 12.40
TC, mmol/L	3.91 ± 1.31
TG, mmol/L	1.40 ± 1.13
HDL, mmol/L	0.96 ± 0.31
LDL, mmol/L	2.42 ± 0.87
Cr, mmol/L	108.75 ± 79.30
Hemoglobin, g/L	134 ± 22
Potassium, mmol/L	4.16 ± 0.52
Sodium, mmol/L	139.46 ± 4.10
NT-proBNP (pg/mL)	3,750 (1,555–8,645)
SBP, mmHg	127 ± 24
DBP, mmHg	81 ± 17
Hypertension^a^	392 (39.2%)
Diabetes^a^	175 (17.5%)
Hperlipidemia^a^	50 (5%)
Current smoking^a^	390 (39%)
ACEI^a^	468 (46.8%)
ARB^a^	55 (5.5%)
Spironolactone^a^	398 (39.8%)
β-blocker use^a^	435 (43.5%)

*Data are expressed as means ± SD or percentages.*

*TC, total cholesterol; TG, triglyceride; HDL, high density lipoprotein cholesterol; LDL, low density lipoprotein cholesterol; Cr, creatinine; SBP, systolic blood pressure; DBP, diastolic blood pressure; ACEI, angiotensin-converting enzyme inhibitor; ARB, angiotensin receptor blockers.*

a *Listed as number (%)*.

### Differential Gene Expression Analysis

**Figure 1:**
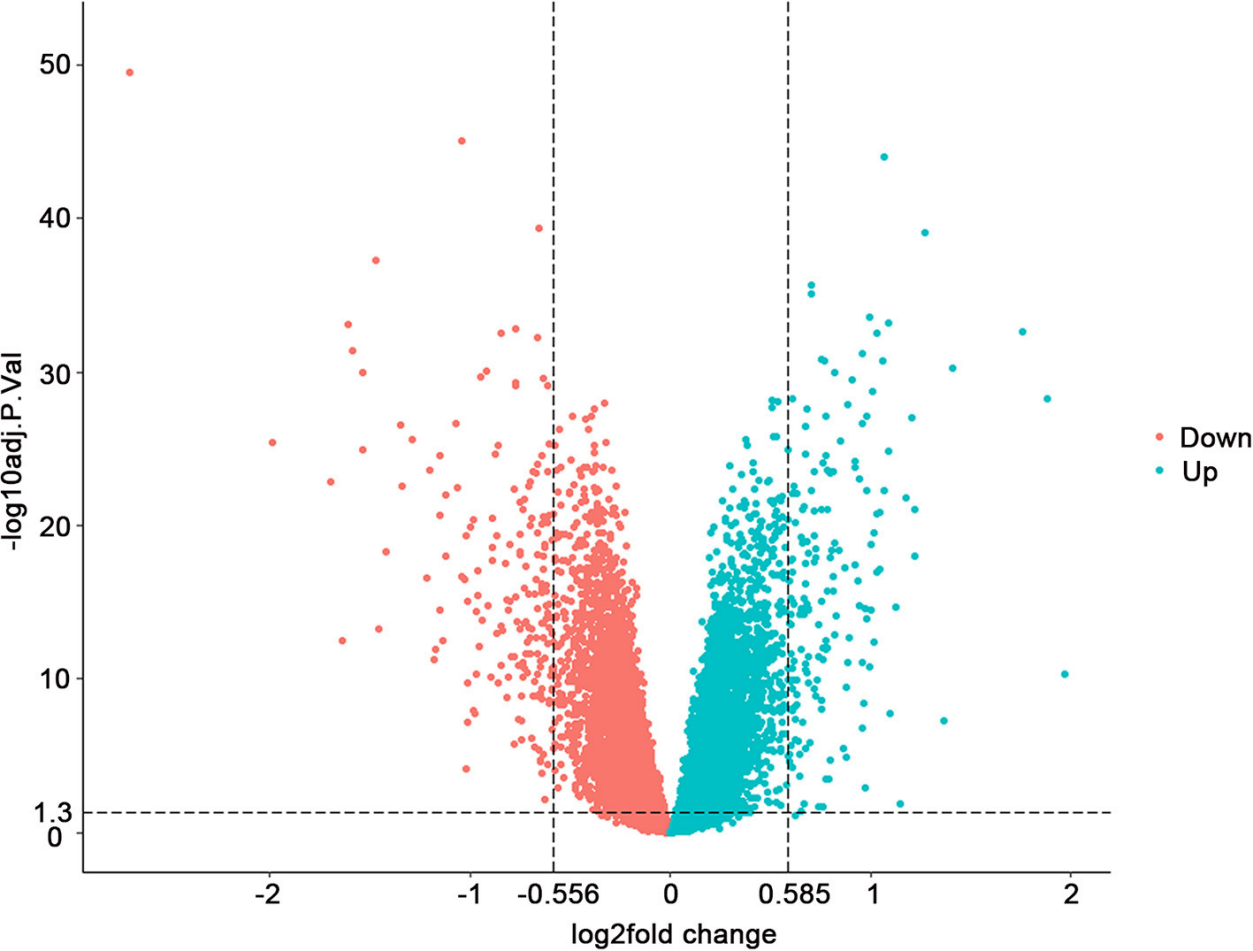
Differential gene expression between 177 failing hearts and 136 healthy donor controls. Volcano plots depicting the extent (x-axis) and significance (y-axis) of differential gene expression between failing and healthy heart samples. Fold change represents failing vs. control hearts.

Through analyzing the transcriptomic data from GEO (accession number GSE57338), we found 157 upregulated and 173 downregulated genes with adjusted *P* < 0.05 when the threshold of FC was set at >1.5 and <0.68 (Supplementary Table 1). The FDR (false discovery rate), which could reduce the false positive rate, was used for the adjustment of the *p-*value. The overview of the comparison of the differential gene expression between HF and control groups is shown in Figure 1.

### SNP Prognosis Analysis

A total of 582 common SNPs in the above selected 330 differential expression genes were found from our WES data. Subsequently, we performed Kaplan–Meier curve analysis for 582 variants using our follow-up data. A total of 37 and 6 SNPs were associated with the prognosis of HF in the dominant (Supplementary Table 2) and recessive models (Supplementary Table 3), respectively. Given that rs420137, rs436743, rs370434, rs420054, rs404435, rs3003174, rs402388, rs2501176, and rs2932988 were in strong LD (r^2^ > 0.9) with each other, we selected rs420137 as the tagged SNP for further GRS development. Similarly, rs741143, rs3210140, and rs653521 were, respectively, chosen as tagged SNPs for their LD with other SNPs (Supplementary Figure 1). Although rs2297224 showed statistical significance in both the dominant and recessive models, we regarded it as a recessive model since it has a smaller *P-*value and higher HR. Finally, 27 SNPs in the dominant model (Table 2) and 4 SNPs in the recessive model (Table 3) were prepared to develop the GRS.

**Table 2:** Statistically significant variants using Cox proportional hazard analysis in dominant model.

**SNPs**	**Mapped genes**	**Function**	**Allele**		**Risk allele**	**MAF**	***P-*value**	**HR**
			**Minor**	**Major**				
rs1715919	MNS1	Missense	G	T	G	0.067	0.000707	1.71
rs11083543	FCGBP	Missense	G	C	G	0.216	0.001803	1.47
rs61761894	SFRP4	Synonymous	T	C	T	0.177	0.002206	1.47
rs741164	C16orf89	Synonymous	C	T	C	0.439	0.005191	1.51
rs420137	FNDC1	Missense	C	G	C	0.354	0.005454	1.43
rs3738530	NID1	Synonymous	A	T	A	0.062	0.005787	1.58
rs16946429	NUDT7	Missense	G	A	G	0.135	0.006227	1.44
rs3169983	SERPINB8	Missense/3UTR	G	A	G	0.176	0.007421	1.40
rs10961757	FREM1	Synonymous	A	G	G	0.451	0.012142	1.39
rs948847	APLNR	Synonymous	C	A	A	0.288	0.014759	1.36
rs3817602	GLT8D2	Synonymous	T	C	C	0.15	0.015021	1.43
rs423490	C3	Synonymous	T	C	T	0.07	0.018452	1.48
rs1802074	SFRP4	Missense	A	G	A	0.245	0.018636	1.34
rs1463725	MED12L	Synonymous	C	T	C	0.317	0.024048	1.32
rs741143	FCGBP	Missense	C	T	C	0.442	0.024293	1.38
rs1869608	MATN2	Synonymous	G	A	A	0.138	0.026984	1.40
rs17221959	SLC11A1	Synonymous	T	C	T	0.1	0.0318	1.37
rs9370340	FAM83B	Synonymous	C	T	T	0.064	0.03354	1.62
rs1981529	STEAP4	Missense	G	A	G	0.114	0.033187	1.35
rs35179634	RAB15	Missense/Synonymous	G	T	G	0.488	0.039924	1.36
rs61748727	P2RX5	Missense	A	G	G	0.059	0.043462	1.60
rs2229682	SLC2A1	Synonymous	A	G	A	0.088	0.04386	1.36
rs6227	FURIN	3UTR	T	C	C	0.069	0.047261	1.51
rs1351113	KLRK1	3UTR	A	G	A	0.119	0.046709	1.32
rs638551	FNDC1	Synonymous	A	G	G	0.406	0.048468	1.28
rs2269287	EDIL3	Synonymous	A	G	G	0.125	0.048747	1.36
rs35016536	LAD1	Frameshift	G	GC	G	0.055	0.049257	1.43

*SNPs, single nucleotide polymorphisms; MAF, minor allele frequency; HR, hazard ratio; UTR, untranslated region*.

**Table 3:** Statistically significant variants using Cox proportional hazard analysis in recessive model.

**SNPs**	**Mapped genes**	**Function**	**Allele**		**Risk allele**	**MAF**	***P-*value**	**HR**
			**Minor**	**Major**				
rs2297224	TUBA3C	Synonymous	A	G	G	0.417	0.006068	1.7
rs3210140	CD163	Synonymous	C	T	T	0.372	0.007582	1.8
rs653521	FNDC1	Synonymous	T	C	C	0.406	0.01784	1.57
rs10733289	FREM1	Synonymous	T	C	C	0.329	0.035378	1.72

### GRS

**Figure 2:**
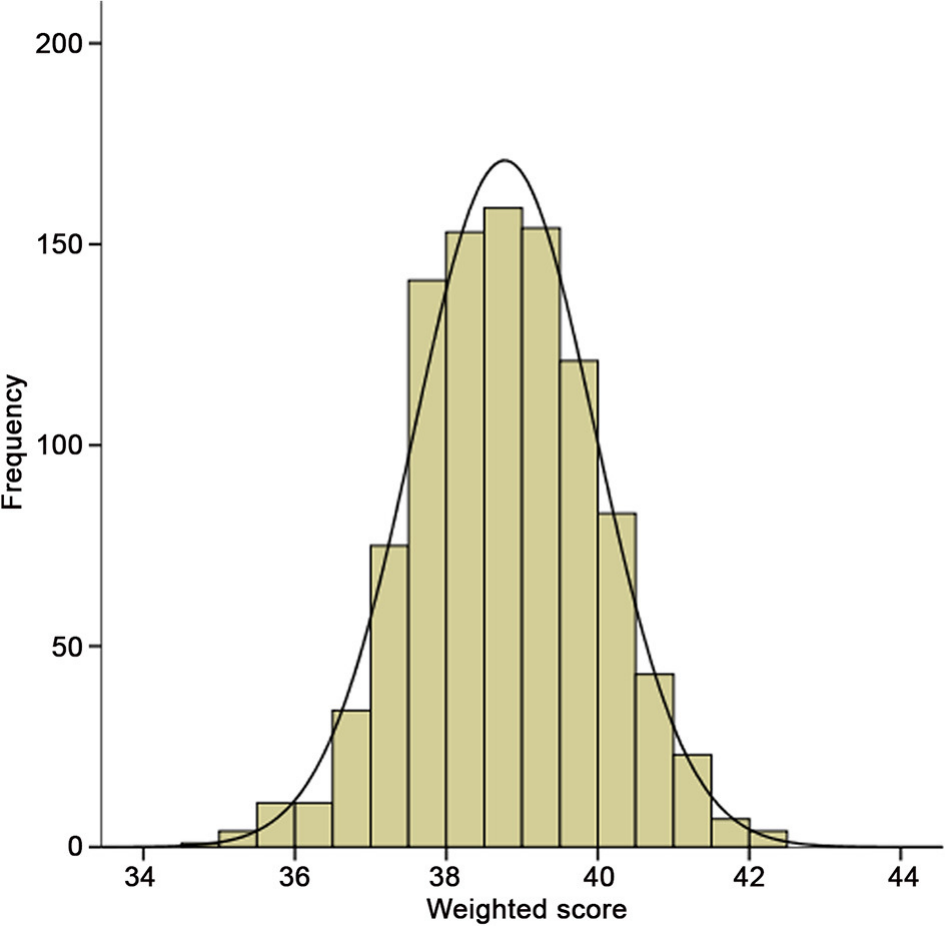
Distribution of integer risk score for all 998 HF patients. The distribution shows a nearly bell-shaped curve, ranging from 34.82 to 42.23 points with a median value of 38.78.

To evaluate the cumulative effects of above 31 SNPs, GRS for each individual was calculated. As shown in Figure 2, the GRS conformed to a bell-shaped distribution, ranging from 34.82 to 42.23 points with a median value of 38.78. We divided the scores into thirds of low (34.82–38.20), medium (38.21–39.26), and high (39.27–42.23) risk from the overall GRS. These accounted for 33.4, 33.4, and 33.2% of chronic HF patients and 11.5, 29.1, and 59.4% of primary endpoint events, respectively. The baseline characteristics of the participants in the low-, medium-, and high-risk groups are listed in Table 4.

**Table 4:** Baseline clinical characteristics of the study cohort with different risk score.

**Characteristics**	**GRS**			***P-*value**
	**Low risk score (*N =* 333)**	**Medium risk score (*N =* 333)**	**High risk score (*N =* 332)**	
Age (years)	55.59 ± 14.92	56.51 ± 14.46	58.89 ± 12.98	0.008
Male, %	75	74	74	0.931
HBP, %	44	36	38	0.082
Diabetes, %	17	17	19	0.769
Current smoker, %	37	41	27	<0.001
β-blocker use	48	45	35	0.002
Ejection fraction, %	35.97 ± 13.57	33.60 ± 11.65	33.93 ± 11.56	0.029
Systolic blood pressure (mmHg)	132.39 ± 61.15	128.01 ± 24.70	125.01 ± 23.99	0.062
Diastolic blood pressure (mmHg)	82.48 ± 18.58	80.58 ± 16.09	78.87 ± 16.66	0.026
Hemoglobin (g/L)	117.26 ± 41.09	122.55 ± 38.19	120.30 ± 36.75	0.374
Creatinine (mmol/L)	97.49 ± 41.22	94.98 ± 33.16	97.10 ± 39.62	0.294
Sodium (mmol/L)	139.18 ± 4.33	139.53 ± 3.85	139.47 ± 3.66	0.584
NT-proBNP (pg/mL)	2,670 (938–6,521)	2,985 (1,479–8,634)	3,866 (1,260–9,000)	0.007

*All continuous variables are expressed as mean ± SD, or median (25th−75th percentile) for right-skewed data.*

*HBP, high blood pressure*.

**Figure 3:**
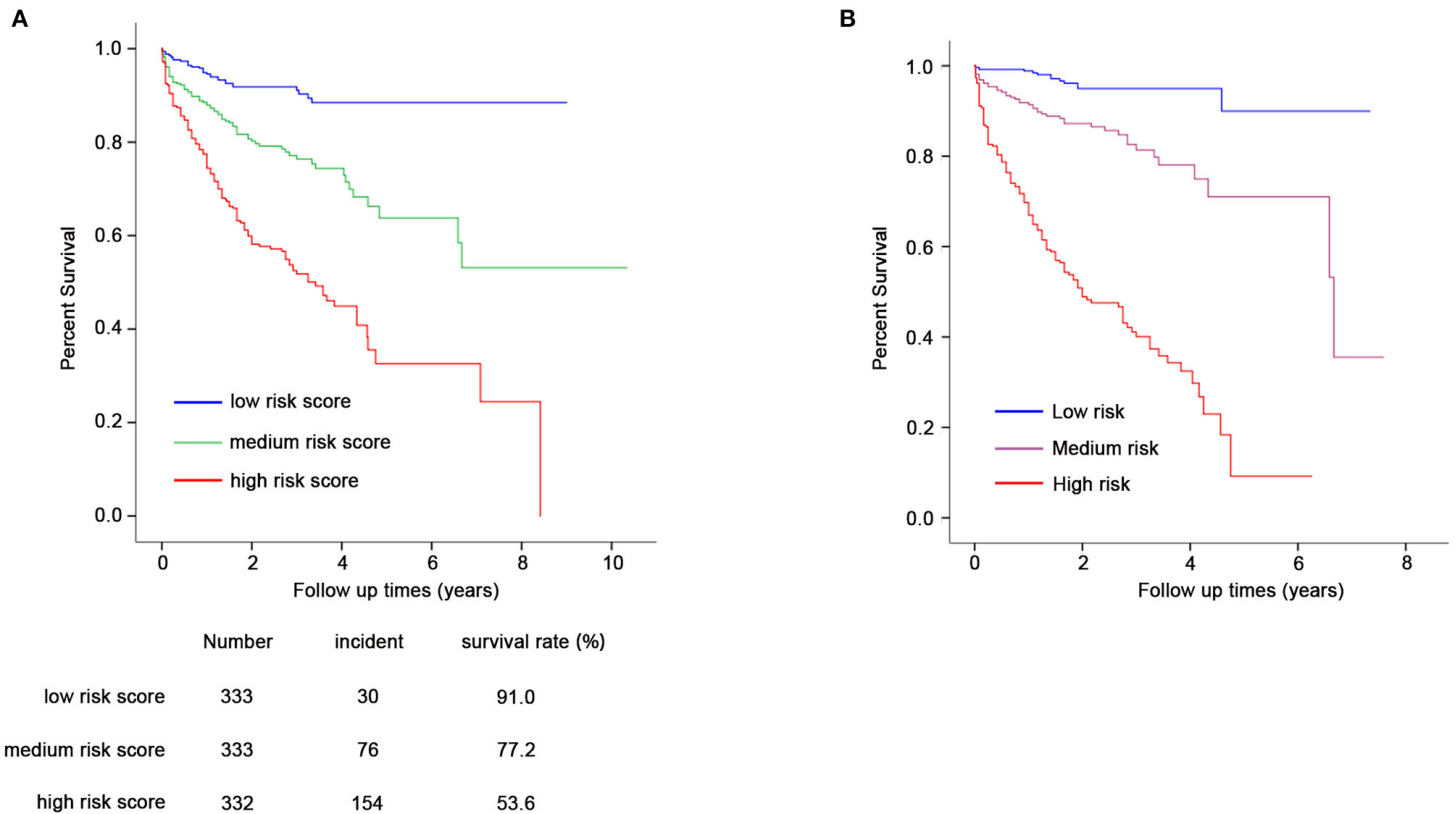
Prognostic analysis for GRS and composite risk score **(A,B)**. Cox proportional hazards regression model was used for prognosis analysis. **(A)** Compared with the low-risk group (*N* = 333), medium (*N* = 333), and high-risk groups (*N* = 332) showed increased HF-related mortality risk (HR = 2.78, 95% CI = 1.82–4.24, *P* = 2 × 10^−6^ for medium- vs. low-risk group; HR = 6.54, 95% CI = 4.42–9.71, *P* = 6 × 10^−21^ for high- vs. low-risk group). The statistical significance remains after adjustment for age, gender, hypertension, diabetes, hyperlipidemia, smoking status, and β-blocker use. **(B)** Composite risk score with medium and high risk showed significantly increased mortality risk of HF (HR = 5.41, 95% CI = 2.72–10.64, *P* = 1 × 10^−6^ for medium vs. low risk; HR = 22.72, 95% CI = 11.90–43.48, *P* = 5 × 10^−21^ for high vs. low risk).

Furthermore, we conducted prognosis analysis using the Cox proportional hazards regression model. As shown in Figure 3A, compared with the low-risk group, medium- and high-risk groups were associated with poorer prognosis of HF (HR = 2.78, 95% CI = 1.82–4.24, *P* = 2 × 10^−6^ for medium vs. low risk group; HR = 6.54, 95% CI = 4.42–9.71, *P* = 6 × 10^−21^ for high vs. low risk group) (Table 5). The statistical significance in multivariate analysis remained after adjusting for traditional risk factors including age, gender, hypertension, diabetes, hyperlipidemia, smoking status, and β-blocker use (HR = 2.38, 95% CI = 1.55–3.66, *P* = 7 × 10^−5^ for medium vs. low risk group; HR = 5.43, 95% CI = 3.65–8.06, *P* = 6 × 10^−17^ for high vs. low risk group) (Table 5).

**Table 5:** Prognosis analysis for groups with different risk score using Cox proportional hazards regression model.

**Groups**	**Unadjusted**			**Adjusted**		
	***P***	**HR**	**95% CI**	***P***	**HR**	**95% CI**
Low-risk score	Reference	Reference	Reference	Reference	Reference	Reference
Medium-risk score	2 × 10^−6^	2.78	1.82–4.24	7 × 10^−5^	2.38	1.55–3.66
High-risk score	6 × 10^−21^	6.54	4.42–9.71	6 × 10^−17^	5.43	3.65–8.06

*The p-value was adjusted with traditional risk factors including age, gender, hypertension, diabetes, hyperlipidemia, smoking status, and β-blocker use*.

### Composite Risk Score

Traditional risk variables were in combination with GRS for the evaluation of the composite effect. After multivariable Cox proportional hazards analysis with all HF mortality-related traditional risk factors and GRS, there remained 10 variables that showed significant association with the prognosis of HF (Table 6). As shown in Table 6, all continuous and categorical variables have respective beta coefficients, which were weighted for composite risk score construction. The low, medium, and high risk of the composite risk score accounted for 5.1, 23.9, and 71.0% of primary endpoint events, respectively.

**Table 6:** Cox regression analysis of association between HF-related mortality risk and continuous variables categorized into groups of low, medium, and high.

**Variable**	**HR**	**CI (95%)**	**Beta coefficient**	***P***
Male sex^a^	1.35	1.03–1.76	0.301	0.026
Diabetes mellitus^a^	1.56	1.06–2.30	0.443	0.025
LVEF^b^ (%)				
Low (39–76)	1.0(Ref)	NA	NA	NA
Medium (29–38)	1.74	1.24–2.42	0.552	0.001
High (10–28)	2.06	1.49–2.85	0.722	<0.001
Potassium^b^ (mmol/L)				
Low (2.57–3.93)	1.0(Ref)	NA	NA	NA
Medium (3.94–4.30)	1.13	0.80–1.59	0.122	0.48
High (4.31–6.91)	1.60	1.17–2.20	0.472	0.004
Sodium^b^ (mmol/L)				
Low (141.2–198.3)	1.0(Ref)	NA	NA	NA
Medium (138.6–141.1)	1.41	0.97–2.05	0.345	0.069
High (114.3–138.5)	2.59	1.85–3.63	0.953	<0.001
NT-proBNP^b^ (pg/mL)				
Low (3.69–1,920)	1.0(Ref)	NA	NA	NA
Medium (1,921–5,757)	3.33	2.09–5.32	1.205	<0.001
High (5,758–79,000)	7.09	4.57–10.99	1.957	<0.001
Age^b^ (years)				
Low (13–52)	1.0(Ref)	NA	NA	NA
Medium (53–65)	1.45	1.04–2.02	0.373	0.03
High (66–94)	2.30	1.68–3.15	0.834	<0.001
DBP^b^ (mmHg)				
Low (86–198)	1.0(Ref)	NA	NA	NA
Medium (73–85)	1.14	0.82–1.59	0.133	0.433
High (40–72)	2.11	1.56–2.86	0.747	<0.001
Cr^b^ (mmol/L)				
Low (32–79)	1.0(Ref)	NA	NA	NA
Medium (80–102)	1.02	0.74–1.41	0.019	0.91
High (103–677)	1.49	1.11–2.01	0.4	0.009
GRS^b^				
Low (34.82–38.20)	1.0(Ref)	NA	NA	NA
Medium (38.21–39.26)	2.78	1.82–4.24	1.022	<0.001
High (39.27–42.23)	6.54	4.42–9.71	1.877	<0.001

*GRS, genetic risk score.*

a *Yes/no.*

b *Divided into groups of low, medium, and high*.

Prognostic analysis using the Cox proportional hazards regression model showed that the composite risk scores with medium and high risk were significantly associated with increased mortality risk of HF when compared with low risk (HR = 5.41, 95% CI = 2.72–10.64, *P* = 1 × 10^−6^ for medium vs. low risk; HR = 22.72, 95% CI = 11.90–43.48, *P* = 5 × 10^−21^ for high vs. low risk) (Table 7, Figure 3B).

**Table 7:** Prognostic analysis for composite risk score using Cox proportional hazards regression model.

**Group**	***P***	**HR**	**95% CI**
Low risk	Reference	Reference	Reference
Medium risk	1 × 10^−6^	5.41	2.72–10.64
High risk	5 × 10^−21^	22.72	11.9–43.48

### Discriminative Power Analysis

**Figure 4:**
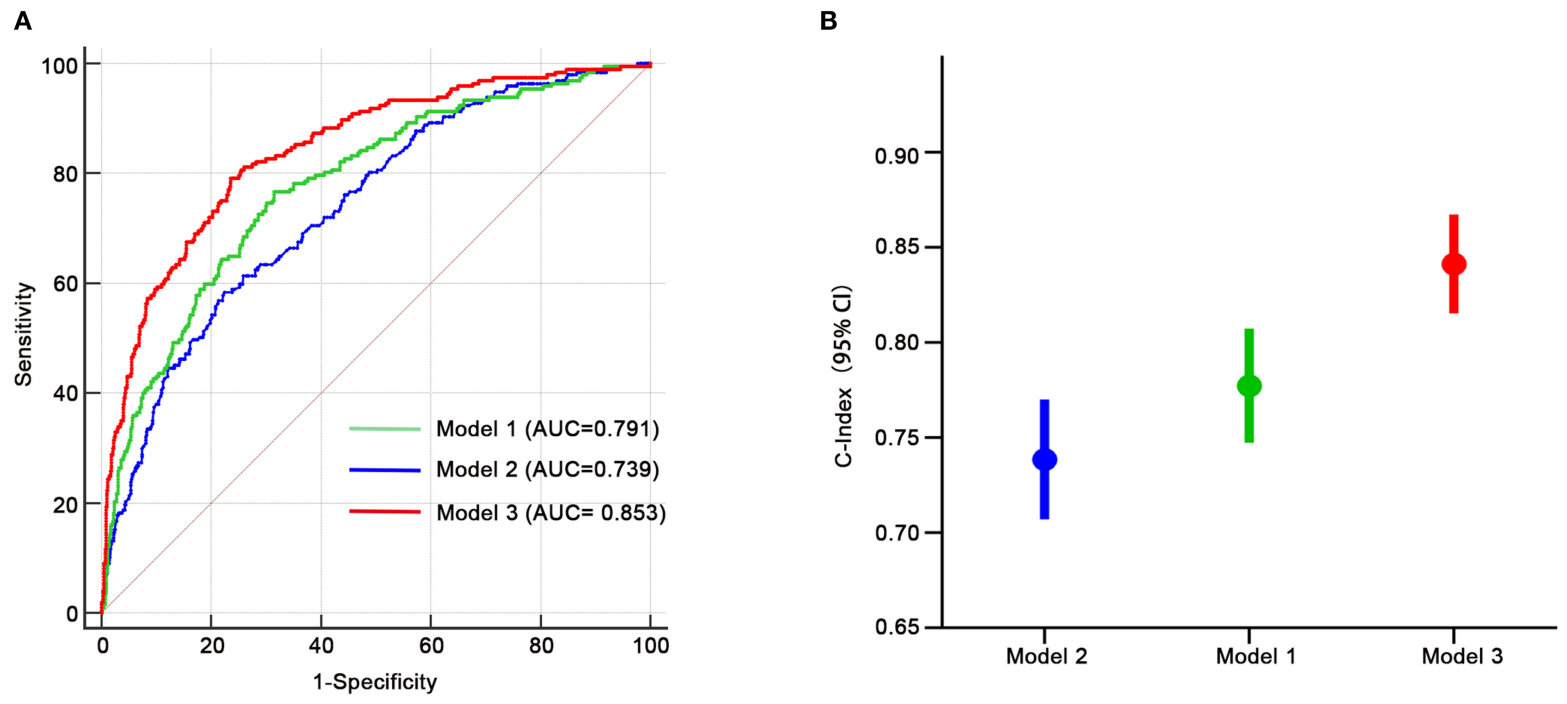
Receiver-operating characteristic curves for HF-related mortality risk. **(A,B)** Model 1, only age, gender, diabetes, LVEF, log-transformed NT-proBNP, serum creatinine, sodium, potassium, diastolic blood pressure; model 2, only GRS; model 3, composite risk score. AUC, area under the curve.

We assessed the discriminative power of the three models: model 1, nine traditional risk factors (TRFs) only; model 2, GRS; model 3, composite risk score. The average AUCs for models 1, 2, and 3 were 0.791 (95% CI = 0.761–0.819), 0.739 (95% CI = 0.707–0.770), and 0.853 (95% CI = 0.826–0.877), respectively. Their true prediction rates reached up to 79.3, 75.4, and 83.5%, respectively. The ROC curves for the three models are shown in Figure 4A. There was no statistically significant difference between models 1 and 2 (*P* = 0.06). However, the composite risk score could significantly improve the discriminative power when compared with TRF or GRS alone (*P* < 0.0001 for model 3 vs. model 1; and *P* < 0.0001 for model 3 vs. model 2) (Figure 4B). In order to avoid overfitting, we conducted cross-validations. The population was randomly divided into two groups, including the training set (449 patients) and the validation set (449 patients). As shown in Table 8, the composite risk score was superior to both TRF and GRS in discriminative power in the training and validation sets (training set: *P* < 0.0001 for model 3 vs. model 1, and *P* < 0.0001 for model 3 vs. model 2; validation set: *P* = 0.022 for model 3 vs. model 1, and *P* < 0.0001 for model 3 vs. model 2), which is consistent with the results from the total population. Besides, the discriminative power showed no difference between models 1 and 2 (Table 8).

**Table 8:** Discrimination power analysis of three models using cross-validations.

**Groups**	**Training set**			**Validation set**		
	**C-Index**	**95% CI**	***P-*value**	**C-Index**	**95% CI**	***P-*value**
Model 1	0.764	0.719–0.80	Reference	0.793	0.749–0.832	Reference
Model 2	0.749	0.703–0.791	0.678	0.727	0.679–0.771	0.124
Model 3	0.841	0.801–0.875	<0.0001	0.842	0.802–0.877	0.022

*P-values were calculated with reference to model 1*.

## Discussion

Our results indicated that medium- and high-risk score groups were associated with 2.78- and 6.54-fold higher mortality risk when compared with the low-risk score group (HR = 2.78, 95% CI = 1.82–4.24, *P* = 2 × 10^−6^ for medium- vs. low-risk group; HR = 6.54, 95% CI = 4.42–9.71, *P* = 6 × 10^−21^ for high- vs. low-risk group). Furthermore, we combined GRS and traditional risk factors to construct the composite risk score, which could more significantly distinguish individuals with different mortality risk (HR = 5.41, 95% CI = 2.72–10.64, *P* = 1 × 10^−6^ for medium vs. low risk; HR = 22.72, 95% CI = 11.90–43.48, *P* = 5 × 10^−21^ for high vs. low risk). Besides, we compared the discriminative power of traditional risk factors, GRS, and combined models for HF using ROC curve analysis. The data showed that GRS and TRF were comparable in the discriminative power (*P* = 0.06), both with a high c statistic beyond 0.7. The combination of TRF and GRS could significantly increase the ability of prediction for survival of HF with c statistic reaching up to 0.853.

Heart failure has been a serious social problem with high mortality (9, 14, 24). Despite advanced drug and device therapies, 5-year mortality rates remained <40% (25, 26). Up to now, a series of HF-related traditional risk factors have been used to construct the prognostic multivariable models for mortality risk stratification (6, 14, 27–31). They all had a well discrimination power with C statistic beyond 0.7 (9, 13, 17). Besides, the prognostic value of circulating microRNAs on the mortality risk of HF has also been investigated recently (32, 33). Importantly, plenty of studies on the association between genetic variants and the prognosis of HF have shed light on the variable mortality risk of individual patients. Based on these, our study was carried out to comprehensively construct a GRS and composite risk score for HF prognosis.

First, our investigation was based on the data from transcriptomic analysis of 313 human heart samples and WES of 998 HF patients, which could comprehensively assess the SNPs associated with HF-related mortality risk.

Second, our GRS was constructed with a total of 31 SNPs, which represented the largest GRS study for the prognosis of HF (18, 22). Furthermore, our GRS achieved greater risk discrimination than the previously published genomic risk score (22). For example, the medium- and high-risk score groups have 2.78- and 6.54-fold HR, respectively, for the prognosis of HF in comparison with the low-risk score group. Importantly, the prediction ability was independent of traditional risk factors. Notably, the composite risk score could dramatically improve the discrimination ability with the mortality risk of high and medium risk reaching up to 22.72- and 5.41-fold, respectively, when compared with the low-risk group. The risk stratification for HF patients could help identify those patients in need of more intensive treatment and also help target appropriate populations for trials of new therapies.

Third, the discriminative power of GRS was displayed excellently, which was comparable to the traditional prediction models with nine known risk factors at present. And the GRS added substantial prognostic power to the traditional risk model with a c-index of 0.853. These suggested that the combination of genetic and traditional risk factors could well discriminate the risk mortality for individual patients, which represented a promising direction in the future.

The main limitation of our study was the single-center study with only one cohort. Although the results were statistically significant, additional larger studies would help confirm our findings.

## Conclusions

In conclusion, we found a total of 31 SNPs associated with HF-related mortality risk by using large-scale prognosis analysis. GRS, derived from the 31 SNPs, was significantly associated with the prognosis of HF and displayed excellent discrimination ability for mortality risk of HF. Moreover, the combination of GRS and conventional risk factors could substantially improve the discrimination power. The results indicated that our GRS could identify individuals with increased HF-related mortality risk and provide clinician with reference for the intensive therapy, especially when combined with traditional risk factors. Future strategies for prognostic assessment of HF should include an individualized assessment in which traditional risk factors are combined with an evaluation of GRS as well.

## Data Availability Statement

The datasets used and/or analysed during the current study are available from the corresponding author on reasonable request.

## Ethics Statement

The studies involving human participants were reviewed and approved by Review Board of Tongji College of Medicine. The patients/participants provided their written informed consent to participate in this study.

## Author Contributions

DH and SH designed experiments. DH, SL, and YS analyzed data. DH and LX wrote the manuscript. DH performed experiments and analyzed data. YW and DW reviewed the manuscript. Sample preparation and protocol were carried out by DH. All authors provided critical feedback and helped shape the research, analysis, and manuscript.

## Conflict of Interest

The authors declare that the research was conducted in the absence of any commercial or financial relationships that could be construed as a potential conflict of interest.

## Abbreviations

HF: heart failure
WES: whole exome sequencing
GRS: genetic risk score
TRF: traditional risk factors
ROC: receiver operating characteristics
SNPs: single nucleotide polymorphisms
MAF: allele frequency
LD: linkage disequilibrium.

## References

[1] Komajda M Charron P Tesson F. Genetic aspects of heart failure. Eur J Heart Fail. (1999) 1:121–6. 10.1016/S1388-9842(99)00026-4 10937920

[2] Li X Zhang P. Genetic determinants of myocardial dysfunction. J Med Genet. (2017) 54:1–10. 10.1136/jmedgenet-2016-104308 27872154

[3] Khan SS Ning H Shah SJ Yancy CW Carnethon M Berry JD. 10-Year Risk Equations for Incident Heart Failure in the General Population. J Am Coll Cardiol. (2019) 73:2388–97. 10.1016/j.jacc.2019.02.057 31097157 PMC6527121

[4] Rossignol P Hernandez AF Solomon SD Zannad F. Heart failure drug treatment. Lancet. (2019) 393:1034–44. 10.1016/S0140-6736(18)31808-7 30860029

[5] Vazquez R Bayes-Genis A Cygankiewicz I Pascual-Figal D Grigorian-Shamagian L Pavon R. The MUSIC Risk score: a simple method for predicting mortality in ambulatory patients with chronic heart failure. Eur Heart J. (2009) 30:1088–96. 10.1093/eurheartj/ehp032 19240065

[6] Jones NR Roalfe AK Adoki I Hobbs FD Taylor CJ. Survival of patients with chronic heart failure in the community: a systematic review and meta-analysis. Eur J Heart Fail. (2019) 21:1306–25. 10.1186/s13643-018-0810-x 31523902 PMC6919428

[7] Pocock SJ Wang D Pfeffer MA Yusuf S McMurray JJ Swedberg KB. Predictors of mortality and morbidity in patients with chronic heart failure. Eur Heart J. (2006) 27:65–75. 10.1093/eurheartj/ehi555 16219658

[8] Yap J Lim FY Chia SY Allen JC Jaufeerally FR Macdonald MR. Prediction of survival in Asian patients hospitalized with heart failure: validation of the OPTIMIZE-HF risk score. J Card Fail. (2019) 25:571–5. 10.1016/j.cardfail.2019.02.016 30822512

[9] Sartipy U Dahlström U Edner M Lund LH. Predicting survival in heart failure: validation of the MAGGIC heart failure risk score in 51,043 patients from the Swedish heart failure registry. Eur J Heart Fail. (2014) 16:173–9. 10.1111/ejhf.32 24464911

[10] Ersbøll M Valeur N Mogensen UM Andersen MJ Møller JE Velazquez EJ. Prediction of all-cause mortality and heart failure admissions from global left ventricular longitudinal strain in patients with acute myocardial infarction and preserved left ventricular ejection fraction. J Am Coll Cardiol. (2013) 61:2365–73. 10.1016/j.jacc.2013.02.061 23563128

[11] Xanthopoulos A Tryposkiadis K Giamouzis G Konstantinou D Giannakoulas G Karvounis H. Larissa heart failure risk score: a proposed simple score for risk stratification in chronic heart failure. Eur J Heart Fail. (2018) 20:614–6. 10.1002/ejhf.1132 29271552

[12] Win S Hussain I Hebl VB Dunlay SM Redfield MM. Inpatient mortality risk scores and postdischarge events in hospitalized heart failure patients: a community-based study. Circ Heart Fail. (2017) 10:e003926. 10.1161/CIRCHEARTFAILURE.117.003926 28701328 PMC5637410

[13] Agostoni P Corrà U Cattadori G Veglia F La Gioia R Scardovi AB. Metabolic exercise test data combined with cardiac and kidney indexes, the MECKI score: a multiparametric approach to heart failure prognosis. Int J Cardiol. (2013) 167:2710–8. 10.1016/j.ijcard.2012.06.113 22795401

[14] Kalogeropoulos AP Georgiopoulou VV Giamouzis G Smith AL Agha SA Waheed S. Utility of the seattle heart failure model in patients with advanced heart failure. J Am Coll Cardiol. (2009) 53:334–42. 10.1016/j.jacc.2008.10.023 19161882

[15] Pocock SJ Ariti CA McMurray JJ Maggioni A Køber L Squire IB. Predicting survival in heart failure: a risk score based on 39 372 patients from 30 studies. Eur Heart J. (2013) 34:1404–13. 10.1093/eurheartj/ehs337 23095984

[16] O'Connor C Fiuzat M Mulder H Coles A Ahmad T Ezekowitz JA. Clinical factors related to morbidity and mortality in high-risk heart failure patients: the GUIDE-IT predictive model and risk score. Eur J Heart Fail. (2019) 21:770–8. 10.1002/ejhf.1450 30919549 PMC6830509

[17] Levy WC Mozaffarian D Linker DT Sutradhar SC Anker SD Cropp AB. The seattle heart failure model: prediction of survival in heart failure. Circulation. (2006) 113:1424–33. 10.1161/CIRCULATIONAHA.105.584102 16534009

[18] Agra RM Gago-Dominguez M Paradela-Dobarro B Torres-Español M Alvarez L Fernandez-Trasancos A. Obesity-related genetic determinants of heart failure prognosis. Cardiovasc Drugs Ther. (2019) 33:415–24. 10.1007/s10557-019-06888-8 31209632

[19] Hu D Huang J Hu S Zhang Y Li S Sun Y. A common variant of RIP3 promoter region is associated with poor prognosis in heart failure patients by influencing SOX17 binding. J Cell Mol Med. (2019) 23:5317–28. 10.1111/jcmm.14408 31148336 PMC6652837

[20] Angermann CE Kaspar M Marx A Kittel-Schneider S Menhofer D Störk S. A functional variant of the neuropeptide S receptor-1 gene modulates clinical outcomes and healthcare utilization in patients with systolic heart failure: results from the interdisciplinary network heart failure (INH) study. Eur J Heart Fail. (2017) 19:314–23. 10.1002/ejhf.706 27990720

[21] Dorsheimer L Assmus B Rasper T Ortmann CA Ecke A Abou-El-Ardat K. Association of mutations contributing to clonal hematopoiesis with prognosis in chronic ischemic heart failure. JAMA Cardiol. (2019) 4:25–33. 10.1001/jamacardio.2018.3965 30566180 PMC6439691

[22] Li S Sun Y Hu S Hu D Li C Xiao L. Genetic risk scores to predict the prognosis of chronic heart failure patients in Chinese Han. J Cell Mol Med. (2019) 24:285–93. 10.1111/jcmm.14722 31670483 PMC6933418

[23] Cordero P Parikh VN Chin ET Erbilgin A Gloudemans MJ Shang C. Pathologic gene network rewiring implicates PPP1R3A as a central regulator in pressure overload heart failure. Nat Commun. (2019) 10:2760. 10.1038/s41467-019-10591-5 31235787 PMC6591478

[24] Rosenberg J Schou M Gustafsson F Badskjaer J Hildebrandt P. Prognostic threshold levels of NT-proBNP testing in primary care. Eur Heart J. (2009) 30:66–73. 10.1093/eurheartj/ehn525 19029123

[25] Wang JJ-C Rau C Avetisyan R Ren S Romay MC Stolin G. Genetic dissection of cardiac remodeling in an isoproterenol-induced heart failure mouse model. PLoS Genet. (2016) 12:e1006038. 10.1371/journal.pgen.1006038 27385019 PMC4934852

[26] Levy D Kenchaiah S Larson MG Benjamin EJ Kupka MJ Ho KK. Long-term trends in the incidence of and survival with heart failure. N Engl J Med. (2002) 347:1397–402. 10.1056/NEJMoa020265 12409541

[27] Alba AC Agoritsas T Walsh M Hanna S Iorio A Devereaux PJ. Discrimination and calibration of clinical prediction models: users' guides to the medical literature. JAMA. (2017) 318:1377–84. 10.1001/jama.2017.12126 29049590

[28] Collier TJ Pocock SJ McMurray JJ Zannad F Krum H van Veldhuisen DJ. The impact of eplerenone at different levels of risk in patients with systolic heart failure and mild symptoms: insight from a novel risk score for prognosis derived from the EMPHASIS-HF trial. Eur Heart J. (2013) 34:2823–9. 10.1093/eurheartj/eht247 23864130

[29] Feola M Testa M Lombardo E Piccolo S Avogadri E Leto L. The prediction of one-year mortality in elderly congestive heart failure patients: a clinical score. Int J Cardiol. (2013) 168:2895–6. 10.1016/j.ijcard.2013.03.170 23643423

[30] Gardner RS Chong KS Morton JJ McDonagh TA. A change in N-terminal pro-brain natriuretic peptide is predictive of outcome in patients with advanced heart failure. Eur J Heart Fail. (2007) 9:266–71. 10.1016/j.ejheart.2006.07.002 17023207

[31] Jackson CE Castagno D Maggioni AP Køber L Squire IB Swedberg K. Differing prognostic value of pulse pressure in patients with heart failure with reduced or preserved ejection fraction: results from the MAGGIC individual patient meta-analysis. Eur Heart J. (2015) 36:1106–14. 10.1093/eurheartj/ehu490 25616644

[32] Bayés-Genis A Lanfear DE Ronde MW de Lupón J Leenders JJ Liu Z. Prognostic value of circulating microRNAs on heart failure-related morbidity and mortality in two large diverse cohorts of general heart failure patients. Eur J Heart Fail. (2018) 20:67–75. 10.1002/ejhf.984 28949058

[33] Masson S Batkai S Beermann J Bär C Pfanne A Thum S. Circulating microRNA-132 levels improve risk prediction for heart failure hospitalization in patients with chronic heart failure. Eur J Heart Fail. (2018) 20:78–85. 10.1002/ejhf.961 29027324

