# Supplementary figures and images for: Prediction of HF-Related Mortality Risk Using Genetic Risk Score Alone and in Combination With Traditional Risk Factors
Source: Front Cardiovasc Med. 2021 Apr 26;8:634966. doi: 10.3389/fcvm.2021.634966 (PMC8107241; doi:10.3389/fcvm.2021.634966)

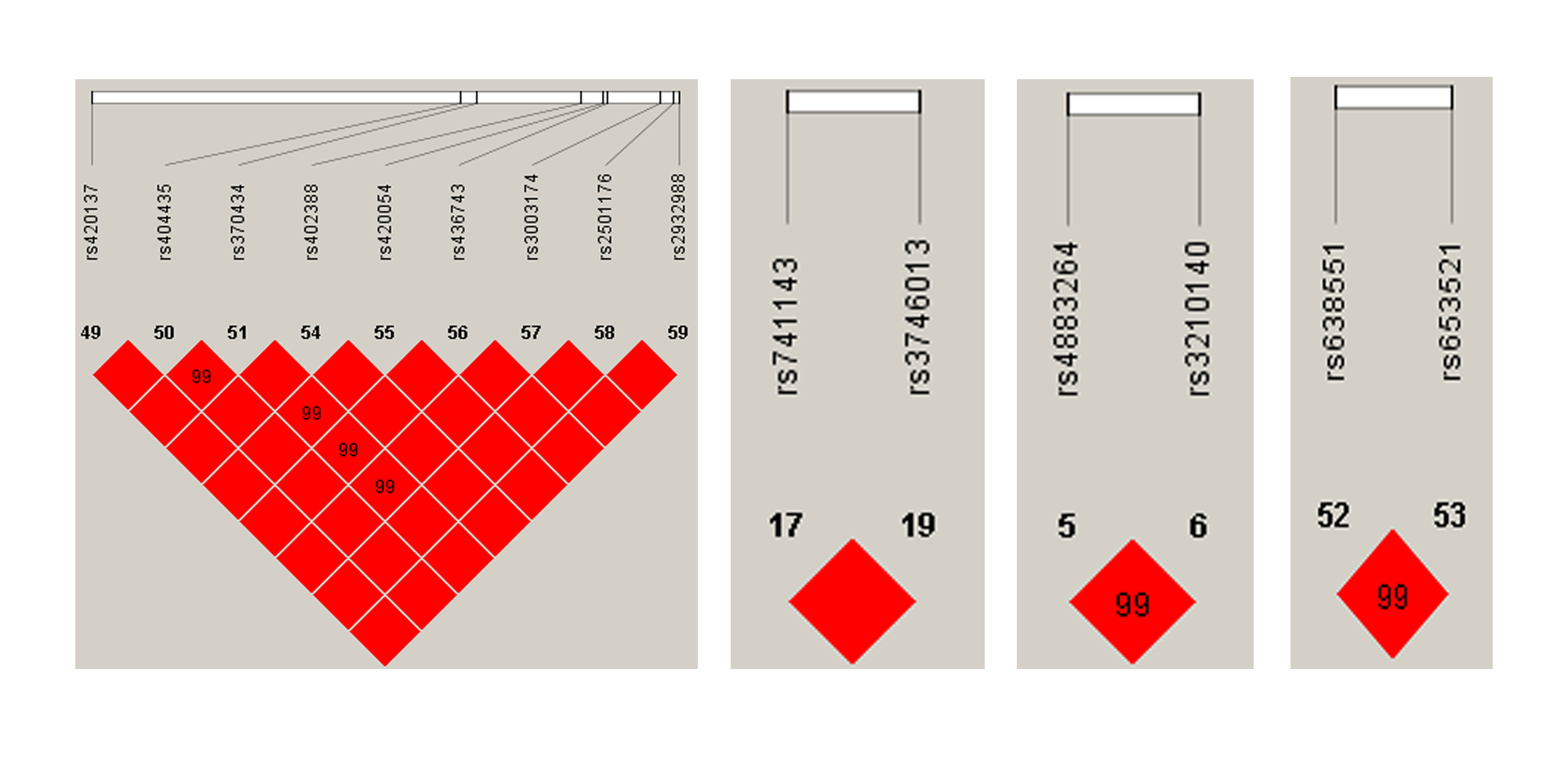

Supplement: Supplementary file 1 [file Image_1.TIF]
